# Overdependence on For-Profit Pharmacies: A Descriptive Survey of User Evaluation of Medicines Availability in Public Hospitals in Selected Nigerian States

**DOI:** 10.1371/journal.pone.0165707

**Published:** 2016-11-03

**Authors:** Boniface Ayanbekongshie Ushie, David Betelwhobel Ugal, Justin Agorye Ingwu

**Affiliations:** 1 Institute of Child Health, College of Medicine, University of Ibadan, Ibadan, Nigeria; 2 Department of Sociology, Federal University Lafia, Lafia, Nigeria; 3 Department of Nursing Sciences, University of Nigeria Nsukka, Nsukka, Nigeria; University College London, UNITED KINGDOM

## Abstract

**Objective:**

Lower availability of medicines in Nigerian public health facilities—the most affordable option for the masses—undermines global health reforms to improve access to health for all, especially the chronically ill and poor. Thus, a sizeable proportion of healthcare users, irrespective of purchasing power, buy medicines at higher costs from for-profit pharmacies. We examined user evaluation of medicine availability in public facilities and how this influences their choice of where to buy medicines in selected states—Cross River, Enugu and Oyo—in Nigeria.

**Methods:**

We approached and interviewed 1711 healthcare users using a semi-structured, interviewer-administered questionnaire as they exited for-profit pharmacies after purchasing medicines. This ensured that both clients who had presented at health facilities (private/public) and those who did not were included. Information was collected on why respondents could not buy medicines at the hospitals they attended, their views of medicine availability and whether their choice of where to buy medicines is influenced by non-availability.

**Principal Findings:**

Respondents’ mean age was 37.7±14.4 years; 52% were males, 59% were married, 82% earned ≥NGN18, 000 (US$57.19) per month, and 72% were not insured. Majority (66%) had prescriptions; of this, 70% were from public facilities. Eighteen percent of all respondents indicated that all their medicines were usually available at the public facilities, most (29%), some (44%) and not always available (10%). Reasons for using for-profit pharmacies included: health workers attitudes (43%), referral by providers (43%); inadequate money to purchase all prescribed drugs (42%) and cumbersome processes for obtaining medicines.

**Conclusions:**

Lower availability of medicines has serious implications for healthcare behavior, especially because of poverty. It is crucial for government to fulfill its mandate of equitable access to care for all by making medicines available and cheap through reviving and sustaining the drug revolving fund scheme and encouraging the prescription of generic drugs in all public health facilities.

## Introduction

It is the responsibility of every nation to provide quality and affordable healthcare services to its people. The government of Nigeria attempts to fulfill this role of health care provision through the primary, secondary and tertiary levels services. However, as Fadare et al [1] have pointed out, there is still a long way to go before Nigeria can provide universal and comprehensive health care for its citizens similar to Western European countries such as France, Germany, Sweden and the UK, where care is typically free at the point of access. Although the private sector also contributes a sizeable portion of health care services, the public health facilities offer the most affordable option for the majority of Nigerians. However, public health facilities are often characterized by inadequacy of supplies including medicines [2]. Medicines account for 20–60% of health spending in developing countries [3, 4, 5], and up to 90% of the population in developing countries purchase medicines through out-of-pocket payments [6, 7, 8, 9], which has impoverishing effects on the people [10]. Medicines make up the largest family expenditure item after food [11] because they are expensive [12], but offer a simple solution to many health problems, provided they are available [13]. Most African countries, including Nigeria, do not have the capacity to produce the medicine that they need [14], depending on foreign drug companies for their medicines. Medicines in Nigeria are therefore imported and distributed through the public and the private sectors.

The provision of medicines by the government comes in the form of public health facilities. Public health care facilities offer the most affordable opportunity for accessing medicines and health services in Nigeria [14]. Low cost public health services are important especially as out-of-pocket expenditure accounts for up to 70% of health financing [15] in a country where it is estimated that 70.8% of the people live below the poverty line of less than US$1 per day [16]. The national health insurance scheme, which is a viable leeway for people to access health services, covers only those working for the government and organized private sector while the majority of Nigerians are excluded. Other government interventions such as the Drug Revolving Fund (DRF) scheme, which was set up in 1988 following the recommendation of the WHO to guarantee a reliable supply of low cost generic drugs for primary health care centers, do not seem to be yielding the required benefits. After the initial wave of success associated with the DRF scheme, sustaining the benefits of the essential drugs program became difficult because of many issues including of stock-out syndrome, and as a consequent, people’s confidence in the orthodox medical services outlets wavered [17].

The opportunity to utilize affordable public health services is affected by lower availability of medicines in public compared to private health facilities [11, 18], poor access to available medicines [19, 20] and other individual-level variables such as income, education, awareness and knowledge. Lower availability of medicines in public health facilities undermines the global health reforms currently undertaken by countries to improve access to health services for all categories of people, especially the chronically ill and those disadvantaged through limited resources or poverty.

Medicines availability forms one of the most important elements in the quality of health care, and improves the utilization of health services [12, 21, 22]. Consequent on the lower availability of medicines, it has been observed that health care users in government health facilities often obtain their drugs from for-profit private pharmacies. Thus, lower availability of medicines can substantially impedes universal and equitable access to health care in Nigeria.

The private sector comprises private hospitals, a minority of qualified pharmacists and a large proportion of illegal and unqualified medicine peddlers without the most minimal training [14, 23, 24]. Although the private sector contributes a substantial proportion to the overall health delivery in Nigeria [25], their services are mainly for-profit. Many times, only people of substantial means can afford the services in the private hospitals. Health expenditure in the private sector may be catastrophic and may threaten the financial capacity of many Nigerians to maintain an acceptable level of subsistence.

At the same time, people grappling with poverty still purchase drugs at high cost from private pharmacies. This situation raises a number of issues requiring empirical investigation. This study therefore examined how health users evaluate availability of medicines in public health facilities and why they purchase their medicines from for-profit private pharmacies. Previous research in Nigeria on the issue of medicines availability and utilization has focused on training of Patent Medicine Vendors, PMVs [26], Knowledge Attitude and Practice (KAP) of PMVs, drugs supply and medicine quality [27], interaction between patients and PMVs/pharmacists [28, 29], among others. There is a dearth of research on how clients evaluate medicine availability in public facilities. The objectives of the study were, thus, to assess users’ perception of availability of medicines in public health facilities and find out if that perception influences where they purchase their medicine. A study of this nature became necessary in Nigeria because of the huge population in need of health care services, the concerns about the appropriate dispensing of medicines both prescribed and over-the-counter, and affordability issues. Findings from this study provide the basis for potential national measures and initiatives in the future if concerns about the lower availability of medicines are found to affect the utilization of health care.

## Methods

Data were drawn from a larger cross-sectional survey on Medicine Availability in Nigeria (MAIN) carried out from May to July in 2015. The exit technique was utilized to approach and interview healthcare users at the point of purchase of drugs at private and for-profit pharmacies. This approach made it possible for both buyers who had presented at health facilities (private/public) and those who did not, to be included in the survey. Using an interviewer-administered questionnaire (S1 File), data were collected on why respondents were buying their medicines at the private pharmacies instead of at the hospitals where they attended (if they attended), their views on if medicine availability and/or otherwise influenced their choice of where to purchase medicines.

The study was carried out in three states in southern Nigeria. One state each was randomly selected from the three geopolitical zones in the southern part of Nigeria. The states were Cross River (South-south), Enugu (Southeast) and Oyo (Southwest). Participants included all clients who presented to purchase medicines at the pharmacies irrespective of their social or occupational status. So they could have been patients and/or their caregivers and even health care personnel purchasing drugs for their patients.

To select the pharmacies from where participants were recruited, we used purposive sampling technique to select the pharmacies and consecutive recruitment of participants. Registered pharmacies in the state capitals were listed and 10 purposively selected in each state based on the observed size and flow of buyers. These pharmacies were used as points from which health users were recruited and interviewed. Selection of the participants was through a consecutive recruitment of all consenting adult clients patronizing the selected pharmacies during a period of three months. The sample size was determined using the Leslie Kish formula for estimating a single proportion, which is given as: N = (Zab)^2^p(1-p)/d^2^. Where: N = required sample size; Za/b = Z-scores corresponding to a one-sided test = 1.96; P = Population proportion of the outcome measure; and d = acceptable margin of error = 0.02. Thus, assuming a 50% percent prevalence (of health users who purchase medicines from for-profit pharmacies), for a power of 90%, a significance level of 5% and a margin of error of 0.02, the minimum required sample is 2401.

Trained research assistants interviewed every consenting health-user as they exited the pharmacies after purchasing their medicines. After a period of three months of data collection, a total of 1891 participants were interviewed, but due to uncompleted and unusable copies of the questionnaire, the sample included in this analysis was 1711. Overall, a response rate of 79% was attained but with the unusable copies of the questionnaire, this rate dropped to 71%. We accepted this as high enough for our needs given that the margin of error for estimating the sample was set at 0.02 (guaranteed to provide a higher sample size) instead of the usual 0–05 favored in the social sciences. A semi-structured interviewer-administered questionnaire, designed and pretested for content validity and reliability, was used to collect information from patients. The questionnaire sought data on users’ choice of where to buy drugs, why they buy from those sources and their experiences with obtaining drugs from the hospitals. The data (see S2 File) were prepared in Epidata and analysis performed with the aid of the Statistical Package for the Social Sciences (SPSS) version 17.0. Descriptive analysis was performed. Ethical approval was obtained from the University of Ibadan/University College Hospital, Ibadan joint Institutional Review Board with approval number UI/EC/12/0365. Both written and verbal consenting procedures were approved by the ethics committee, based on the knowledge about the cynicism and suspicion regarding the signing of documents. Thus, while some participants provided written informed consent, others who refused signed documentation provided verbal consent. On occasions when verbal consent was provided, the research assistants would sign the consent form and write “participant has read (has been read to) sufficient information concerning the study, and has verbally consented to participate”.

## Results

Table 1 summarizes information on respondents’ socio-demographic characteristics. On the whole, more respondents were interviewed in Oyo state (44.7%) than Enugu and Cross River states. The mean age of participants was 37.7±14.4 years; 40.4% were forty years or older, and only 6.7% were aged 15–19 years; about half (51.6%) of the respondents were men; nearly six in ten participants (58.8%) were married. Some of the respondents were engaged in managerial and technically-inclined occupations (18.4%), but the highest proportion was unskilled workers (34.6%), while students accounted for 25.0%. A majority (82.0%) earned monthly incomes close to, or more than, the national minimum wage of N18, 000, which is equivalent to US$57.19. Health insurance coverage for individuals under the National Health Insurance Scheme (NHIS) was only reported among 27.9% of the participants, another 22.0% reported being covered under some other type of insurance.

**Table 1:** Distribution of respondents according to socio-demographic characteristics.

Characteristics	Frequency (N = 1711)*	Percent
**Mean age and standard deviation**	**37.7±14.4**	
**Age Groups**		
≤19	107	6.7
20–24	206	12.9
25–29	282	17.6
30–34	194	12.1
35–39	163	10.2
≥40	646	40.4
**Gender**		
Male	883	51.6
Female	828	48.4
**Marital status**		
Single	618	36.1
Married	1006	58.8
Divorce	41	2.4
Separated	20	1.2
Widowed	26	1.5
**Education**		
Primary	83	4.9
Secondary	392	23.1
More than secondary	1223	72.1
**Occupation**		
Professional	106	8.6
Managerial and technical	233	18.4
Non-manual skilled	10	0.8
Manual skilled	52	4.1
Partly skilled	67	5.3
Unskilled	439	34.6
Student	317	25.0
No profession	42	3.3
**Income**		
Less than minimum wage	125	18.0
Minimum wage and above	571	82.0
**Enrolled in National Health Insurance**		
No	1232	72.1
Yes	477	27.9
**Enrolled in other health insurance**		
No	1331	78.0
Yes	376	22.0

*All missing values were excluded from analysis

Table 2 shows the medicine seeking characteristics of the participants. For the illness that participants were buying the medicines, 65.7% of them had presented at a health facility before proceeding to the pharmacy to buy medicines; of this proportion, 65.2% reported that they initially attended a government hospital and the rest from a private hospital. Moreover, 17.1% of respondents were purchasing the medicines for either themselves or relatives on admission in a government hospital. Furthermore, 69.7% had a hospital prescription or one from a doctor, while the remaining 30.3% were engaging in self-directed purchases without prescriptions from qualified personnel.

**Table 2:** Distribution of respondents according to whether they presented at a hospital and had prescriptions.

Variable	Frequency (N = 1711)*	Percent
**Prior hospital presentation before pharmacy**		
No	556	34.3
Yes	1064	65.7
**Type of Hospital attended**		
Government	695	66.1
Private	357	33.9
**Person medicines being bought for**		
Self	1104	71.9
Someone else	431	28.1
**Ill person on admission**		
No	1281	82.9
Yes	265	17.1
**Have prescription**		
With hospital/doctor’s prescription	1084	69.7
Buying on my own	471	30.3

*All missing values were excluded from analysis

Data also revealed that 81.1% of participants reported the presence of a government hospital within easy reach of their locality; 18.2% reported no health facility in their neighborhoods; 51% reported a private hospital and 59.3% reported a PMV, commonly referred to as a “chemist”.

Participants were asked to evaluate the availability of medicines in government-owned hospitals where they normally attend when ill (see Table 3 for details). The majority (78.7%) felt that medicines were always available. However, only 17.1% reported that they always had all their medicines available at the government hospitals; 29.3% reported that most of the drugs prescribed for them were available; for 43.8% of the respondents, usually some of the medicines are available and one in ten (9.9%) said medicines were not always available. Further scrutiny of the data revealed that 39.7% of the respondents reported they usually buy some of their medicines outside of the hospital; while 37.3% reported that they usually find some of the medicines to buy in the hospital; only 13.7% reported they could buy all their medicines from the hospital. It was further observed that for those who were (or whose relatives were) on admission in a government facility; slightly more people reported purchasing some of their medicines outside (38.9%) compared with those who were buying in the hospitals (37.4%).

**Table 3:** Respondents’ evaluation of medicine availability and quality in government hospitals.

User evaluation	Frequency	Percent
Medicines often available in that hospital clients usually attend (1635*)		
No	348	21.3
Yes	1287	78.7
Medicines usually available in government hospitals clients have attended (1616*)		
No not at all	160	9.9
Yes all the medicines	276	17.1
Yes most	473	29.3
Yes some	707	43.8
Usually buy all medicines in the hospital (1595*)		
Always buy all in hosp	219	13.7
Some in hosp	595	37.3
Buy all outside	148	9.3
Buy some outside	633	39.7
Opinion of the quality of the medicines in a government hospital (1658*)		
Low standard	308	18.6
Average	682	41.1
High standard	668	40.3
Preferred medicine source based on availability (1668*)		
Government	751	44.7
Private	436	25.9
Chemist	163	9.7
Pharmacy	318	18.9

*All missing values were excluded from analysis

With respect to how the respondents rated the quality of the medicines in government hospitals, 18% felt medicines from government hospitals were substandard and of low quality, 41.1% rated medicines as average while 40.3% rated medicines in government hospital as being of high standard. More than four in ten (44.7%) respondents, however, indicated that they mostly preferred to get medicines from a government hospital; a quarter of the respondent preferred a private hospital (25.7%) or pharmacy (18.9%). Moreover, most respondents (73.2%) indicated they mainly seek care in government facilities when they or members of their households fall ill, 45.9% said they would go to a private hospital; 40.4% said they go first go to a drug store.

Some participants indicated that non-availability of medicines in government hospitals could affect their ability to seek health when such a need arise; 45.6% of the respondents said non-availability of medicines could influence their subsequent choice of seeking health care in a facility that lacks medicines be it government or private. Only 18% said the pharmacy (where they were found and interviewed) was the only place in the community were they could get their medicines and 30.5% of the respondents preferred and could only purchase their medicines from a particular pharmacy. Some (38.5%) of the respondents maintained that the possibility of buying medicines on credit was the sole reason why they buy their medicines at a particular pharmacy (details in Table 4).

**Table 4:** User’s attitude towards medicine non-availability in hospitals.

Outcomes of lower availability of medicines	Frequency (N = 1711)*	Percent
**Non-availability affects ability to seek care**		
No	874	57.8
Yes	637	42.2
**Non-availability influences choice of place of care**		
No	817	54.4
Yes	685	45.6
**Opportunity to obtain medicines on credit reason why user prefers for-profit pharmacy**		
No	381	61.5
Yes	239	38.5
**This is the only source of medicines in the community**		
No	1260	82.0
Yes	276	18.0
**Quality of medicines sold in the for-profit pharmacy**		
Bad	27	1.7
Good	656	41.2
Very good	909	57.1

*All missing values were excluded from analysis

**Fig 1:**
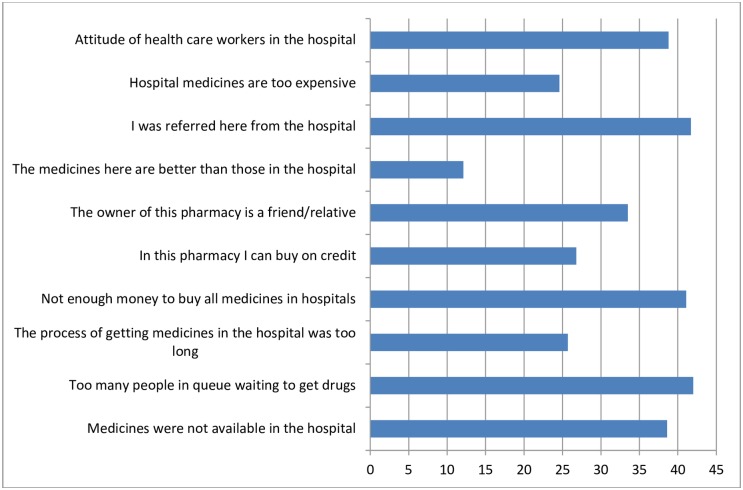
Reasons given by respondents who had prescriptions for using for-profit pharmacies. (n = 1084).

**Fig 2:**
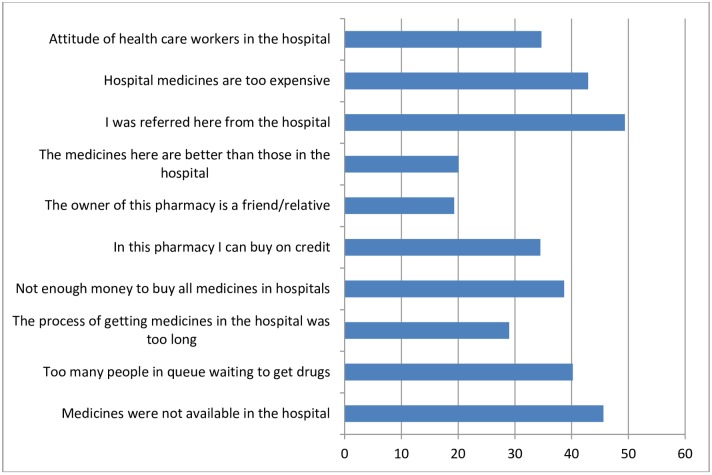
Reasons given by respondents whose relatives were on admission in public facilities for using for-profit pharmacies. (n = 265).

In response to why they purchase medicines from for-profit–pharmacies, some of the reasons given by respondents were not only due to non-availability (see Figs 1 and 2), but mainly due to the fact that many users were being referred by healthcare providers to outside sources for medicine.

## Discussion

A substantial proportion of the sample in this study was those who could earn up to or more than the national minimum wage of NGN18000 (US$57.19) per month. However, with the current state of economic decline and high cost of living, earning an average of US$57 per month, from which all expenditure, including feeding, clothing, children’s education, and health are drawn, may mean living under poverty and deprivation. Thus, having to purchase required medicines from for-profit outfits even after presenting at public health facilities (and possibly paying consultation fees), is in itself indicative of systemic failure, and can prove to be catastrophic for many families. Indeed, it has continued to be argued that the national minimum wage at present is not adequate to take workers home. (This is even for those who have jobs; there are a whole lot of people who have no jobs, neither with the formal nor informal sectors.) To further aggravate this situation, enrolment in the National Health Insurance Scheme is presently tied to employment in the public service or organized private sector, and thus, the majority of Nigerians are not presently having health insurance coverage. The limited earning power and non-membership of the national health insurance have tremendous impact on families’ capabilities to finance health care and still manage to stay afloat.

This study has found availability of medicines to be very poor in public sector facilities, which offer the best options for cheap source of medicines, especially for a majority of low-income-earning Nigerians. This finding is in line with that of Jegede [30] who found out that lack of access to affordable health care services, poor distribution of health care facilities, shortage of drugs, poor attitude of health workers, the enormous cost of health services, which is sometimes out of the reach of the poor, poor infrastructure and poor health education strategy are some of the critical problems of health care delivery in Nigeria. It will appear that the introduction of user-fees to improve medicines availability in public health facilities in Nigeria [31] has not solved the problem of lower availability of medicines in public health facilities. In the study by Uzochukwu and Unwujekwe [31], it was found that at the initial point when the user-fees was introduced along with the Drug Revolving Fund (DRF), there was significant improvement in medicines availability and improvement in health services; however, as the health workers became de-motivated and involved in selling their own drugs for their own profit, the policy failed. Thus, the present finding that an overwhelming proportion of study participants usually do not find their prescribed medicines in the hospitals, only buttresses the notion that Nigerian hospitals are in a decayed state and nothing more than consulting clinics [32, 33]. Nigerian hospitals are referred to as mere consulting clinics because both “free drugs” (as practiced in some states in Nigeria) and even the essential medicines are not available, thus, making the care of common ailments difficult [33]. In circumstances where the majority of the citizenry have to depend on the private sector for their health care services, it means the state has abdicated its key responsibility. This is similar to the position of Alubo [34] when he argued that there are serious limits to depending on private sector health care because the very reason for their existence is profit-making, meaning that while they can provide good services for those who can afford them, private sector health care is unlikely to provide sustainable health services to the majority of Nigerians who live below the poverty line and are underserved.

In this study, it was found that participants’ evaluation of the availability of medicines in public health facilities revealed a particularly poor notion of the availability of medicines in government hospitals. This finding is in consonance with that of Shabangu and Suleman [35] who found most of the respondents in their study were unable to receive all their prescribed medicines in the government hospital in each of their visits in the past six months prior to the study (also see [36, 37, 38]). The finding also resonates with that of Muhammed et al. [39] who found that more than half of the participants (53.6%) in their study preferred PMVs to public health facilities mainly because of the lack of essential medicines and the high cost of care. The patients or their relatives may see public health facilities as costly because, compared to PMVs, hospitals charge for other care services other than medicines whereas at the medicines stores, they can consult the “pharmacists” and only get to pay the cost of the medicines alone.

Given such a reality, and given the fact that patients generally spent a lot more on drugs in the private pharmacies than in government facilities, the financial implication of lower availability of medicines in public facilities becomes considerable and may lead to catastrophic health spending [6, 7]. This is even more so because of the fact that out-of-pocket health expenditure is the more prevalent source of health spending in developing economies [11, 18]. This study, for example, found that the majority of respondents were not covered by any health insurance scheme. Moreover, the national minimum wage, which is the gold standard for remuneration of workers in Nigeria, is equivalent to just about US$57 and if a large proportion of it is devoted to out-of-pocket health expenditure, particularly medicines, then, the low earning Nigerians will be facing dire financial constraints.

As the data have revealed, there are many factors accounting for patients’ inability to obtain their medicines from public health facilities; one of such is the poor attitudes of health workers toward patients. In Uganda, a study has provided ample evidence which collaborate the fact that health workers’ attitude prevent health seekers from patronizing facilities [40]. In Nigeria, studies have found health workers’ attitude to be a major barrier to health utilization [30, 39]. Studies show that the growth of for-profit-pharmacies decreases the availability of health care for "unprofitable" patients. Traditionally, non-profits have financed care for the poor by overcharging paying patients to subsidize services for the poor. For-profits, by refusing to serve non-paying patients while at the same time taking a great share of paying patients, leave non-profits with more of the poor to serve but with fewer paying patients to subsidize their care [41].

From the results, it is also an issue that the procedures for obtaining medicines at public health facilities are cumbersome, which often prompt health users to seek alternatives in private pharmacies where service delivery is prompt and client-friendly. The long procedures for obtaining drugs increase waiting time, which has been found to be an important issue to patients [42, 43]. The cumbersome process, coupled with the unfriendly attitudes of the health care workers in public health facilities have been given as some of the reasons influencing health seeking behaviours (30, 39). For many health users, the process of moving from one part of the hospital to get their prescriptions billed, to pay the bills and then to collect their medicines, is so tiresome and time consuming that they simply prefer to buy at the private pharmacies notwithstanding that medicines are cheaper in public facilities. Also, health care providers often refer patients to buy medicines from private pharmacies. Although these referrals may be given because the drugs are actually not available in the hospitals, in some instances, medical practitioners in public hospitals refer users to outside and privately-owned facilities for treatment or purchase of medications as these facilities are owned by the medical practitioners themselves or their colleagues.

Findings of this study must be interpreted and applied with caution given that the sampling techniques used to select the pharmacies and participants was non-probabilistic, which may impact on the chances of generalization to the whole of Nigeria. Nevertheless, findings reported here are critical issues that should be taken into account in strategizing on how to improve access to medicines, and indeed, health services as a whole. In conclusion, the level of availability of medicines in hospitals influences health care use and choices because health users can become wary of facilities which offer nothing more than consultations. Ensuring that medicines are available and cheap can be an important part of making sure that access to healthcare is improved for all, especially the poor and underserved, in the light of the global drive to achieve equitable access to care.

## Supporting Information

S1 File Study Questionnaire. (DOCX)

S2 File Dataset. (XLSX)

## References

[1] Fadare J. O., Adeoti A. O., Desalu O. O., Enwere O. O., Makusidi A. M., Ogunleye O.,… & Godman B. (2015). The prescribing of generic medicines in Nigeria: knowledge, perceptions and attitudes of physicians. *Expert review of pharmacoeconomics & outcomes research*, 1–12. 10.1586/14737167.2016.1120673 26567041

[2] Bigdeli M., Jacobs B., Tomson G., Laing R., Ghaffar A., Dujardin B. and Van Damme W. Access to medicines from a health system perspective. *Health Policy and Planning*, 2013; 28 (7): 692–704. 10.1093/heapol/czs108 23174879 PMC3794462

[3] WHO. Equitable Access to Essential Medicines: A Framework for Collective Action. Geneva: World Health Organization, 2004a.

[4] Wirtz V. J., Santa-Ana-Tellez Y., Servan-Mori E., & Avila-Burgos L. (2012). Heterogeneous effects of health insurance on out-of-pocket expenditure on medicines in Mexico. *Value in Health*, 15(5), 593–603. 10.1016/j.jval.2012.01.006 22867767

[5] Orem J. N., Mugisha F., Okui A. P., Musango L., & Kirigia J. M. (2013). Health care seeking patterns and determinants of out-of-pocket expenditure for malaria for the children under-five in Uganda. *Malaria journal*, 12(1), 1. 23721217 10.1186/1475-2875-12-175 PMC3680323

[6] WHO. The World Medicines Situation. Geneva: World Health Organization, 2004b.

[7] Garg C. C. and Karan A. K. Reducing out-of-pocket expenditures to reduce poverty: a disaggregated analysis at rural-urban and state level in India. *Health Policy and Planning*, 2009; 24(2): 116–128. 10.1093/heapol/czn046 19095685

[8] Brinda E. M., Andrés R. A., & Enemark U. (2014). Correlates of out-of-pocket and catastrophic health expenditures in Tanzania: results from a national household survey. *BMC international health and human rights*, 14(1), 1. 10.1186/1472-698X-14-5 PMC3946236 24597486

[9] Wang Q., Fu A. Z., Brenner S., Kalmus O., Banda H. T., & De Allegri M. (2015). Out-of-pocket expenditure on chronic non-communicable diseases in sub-Saharan Africa: the case of rural Malawi. *PloS one*, 10(1), e0116897 10.1371/journal.pone.0116897 25584960 PMC4293143

[10] Niëns L. M., Cameron A., Vandepoel E., Ewen M. and Al E. Quantifying the Impoverishing Effects of Purchasing Medicines: A Cross-Country Comparison of the Affordability of Medicines in the Developing World. *PLoS Medicine*, 2010; 7(8): e1000333 10.1371/journal.pmed.1000333 20824175 PMC2930876

[11] Cameron A., Ewen M. Ross-Degnan D. Ball D. and Laing R. Medicine prices, availability, and affordability in 36 developing and middle-income countries: a secondary analysis. *The Lancet*, 2008; 373: 240–249. 10.1016/S0140-6736(08)61762-6 19042012

[12] Odusanya O. O. Drug use indicators at a secondary health care facility in Lagos, Nigeria. *Journal of Community Medicine & Primary Health Care*, 2004; 16(1): 21–24.

[13] Pe´Coul B., Chirac P. Trouiller P. and Pinel J. Access to Essential Drugs in Poor Countries: A Lost Battle? *JAMA*, 1999; 281(4): 361–367. 9929090 10.1001/jama.281.4.361

[14] Foster S. D. *Improving the Supply and Use of Essential Drugs in Sub-Saharan Africa*. Washington DC: The World Bank: Population, Health, and Nutrition Division, Population and Human Resources Department, 1990.

[15] NHA. *National Health Accounts (NHA)*, *2003–2005*. Abuja: National Health Accounts, 2006.

[16] UNDP. Human Development Report. New York: United Nations Development Programme, 2007.

[17] Johnson O. E., Adiakpan N. W., & Asuzu M. C. (2015). Drug availability and health facility usage in a Bamako Initiative and a non-Bamako Initiative Local Government Areas of Akwa Ibom State, South-South Nigeria. *Journal of Community Medicine and Primary Health Care*, 27(2), 73–82.

[18] Mendis S., Fukino K. Cameron A. Laing R and Al E. The availability and affordability of selected essential medicines for chronic diseases in six low- and middle-income countries. *Bulletin of the World Health Organization*, 2007; 85(4): 279–88. 10.2471/BLT.06.033647 17546309 PMC2636320

[19] Salako L. A. Drug supply in Nigeria. *Journal of Clinical Epidemiology*, 2004; 45: 15–19. 10.1016/0895-4356(91)90107-k 2045836

[20] Umoru A., Alfa J., & Adigwe O. P. (2016). Factors Influencing Access to Medicines in Nigeria: Views and Experiences of Residents of the Federal Capital Territory. *Value in Health*, 19(3), A35.

[21] Jitta J., Whyte S. R. and Nshakira N. The availability of drugs: what does it mean in Ugandan primary care. *Health Policy*, 2003; 65(2): 167–179. 12849915 10.1016/s0168-8510(03)00003-4

[22] Odusanya O. O. & Bamgbala A. O. A community based assessment of a model primary health care centre. Nigerian Quarterly Journal of Hospital Medicine, 1999; 9(4): 10.4314/nqjhm.v9i4.12397

[23] Okafor C., & Onuigbo R. A. (2015). Rethinking Public Administration Professionalism in Nigeria. *African Research Review*, 9(4), 333–347.

[24] Iyioha I. O., & Nwabueze R. N. (2016). *Comparative health law and policy*: *critical perspectives on Nigerian and global health law*. Routledge.

[25] FMOH. *Inventory of Health Facilities in Nigeria*. Abuja: Federal Ministry of Health, 2005.

[26] Oshiname F. O. and Brieger W. R. Primary care training for patent medicine vendors in rural Nigeria. *Social Science & Medicine*, 1992; 35(12): 1477–1484. 1485195 10.1016/0277-9536(92)90050-z

[27] Alubo S. O. Death for sale: A study of drug poisoning and deaths in Nigeria.” *Social Science & Medicine*, 1999; 38: 97–103. 10.1016/0277-9536(94)90304-2 8146720

[28] Brieger W. R., Osamor P. E. Salami K. K. Oladepo O. and Otusanya S. A. Interactions between patent medicine vendors and customers in urban and rural Nigeria. *Health Policy and Planning*, 2004; 19: 177–182. 15070866 10.1093/heapol/czh021

[29] Oparah C. A. and Iwuagwu M. A. Public perceptions of community pharmacists in Benin City, Nigeria. *International Journal of Pharmacy Practice*, 2001; 9(3): 191–195.

[30] Jegede A. S. Problems and prospects of health care delivery in Nigeria: issues in political economy and social inequality *Currents and Perspectives in Sociology*, Ibadan: Malthouse Press Limited, 2002; pp.212–226.

[31] Uzochukwu B. & Onwujekwe O. Healthcare reform involving the introduction of user fees and drug revolving funds: influence on health workers’ behavior in southeast Nigeria. *Health Policy*, 2005; 75(1): 1–8. 10.1016/j.healthpol.2005.01.019 16298224

[32] Innocent E. O., Uche O. A. & Uche I. B. Building a solid health care system in Nigeria: challenges and prospects. *Academic Journal of Interdisciplinary Studies*, 2014; 3(6): 501.

[33] Abegunde K. A., & Asuzu M. C. (2013). Facility User's Preference between the Free and the Bamako Initiative (Drug Revolving Fund-Based) Health Services in Iwajowa Local Government, Oyo State. *Journal of Community Medicine and Primary Health Care*, 26(2), 1–6.

[34] Alubo O. The promise and limits of private medicine: health policy dilemmas in Nigeria. *Health Policy Plan*, 2001; 16 (3): 313–321. 11527872 10.1093/heapol/16.3.313

[35] Shabangu K. and Suleman F. Medicines availability at a Swaziland hospital and impact on patients. *African Journal of Primary Health Care and Family Medicine*, 2015; 7(1): 1–6. 10.4102/phcfm.v7i1.829 PMC4656915 26466396

[36] Kotwani A. Where we are now: assessing the price, availability and affordability of essential medicines in Delhi as India plans free medicine for all. *BMC Health Services Research*, 2013; 13: 285–298. 10.1186/1472-6963-13-285 23885985 PMC3733775

[37] Babar Z., Lessing C., Mace C. and Bissell K. The Availability, Pricing and Affordability of Three Essential Asthma Medicines in 52 Low- and Middle-Income Countries. *PharmacoEconomics*, 2013; 31(11): 1063–1082. 10.1007/s40273-013-0095-9 24127259

[38] Sado E. and Sufa A. Availability and affordability of essential medicines for children in the Western part of Ethiopia: implication for access. *BMC Pediatrics*, 2015; 16:40–47. 10.1186/s12887-016-0572-3 PMC4791837 26979737

[39] Muhammed K. A., Umeh K. N., Nasir S. M., & Suleiman I. H. (2013). Understanding the barriers to the utilization of primary health care in a low-income setting: implications for health policy and planning. *Journal of Public Health in Africa*, 4(2), 13. 10.4081/jphia.2013.e13 PMC5345431 28299102

[40] Chandler C. I., Kizito J. Taaka L. Nabirye C. Kayendeke M. DiLiberto D. and Staedke S. G. Aspirations for quality health care in Uganda: how do we get there. *Human Resources for Health*, 2013; 11(1): 13–24. 23521859 10.1186/1478-4491-11-13 PMC3610284

[41] Brock D. W. Buchanan A. E. The Profit Motive in Medicine. Journal of Medicine and Philosophy, 1987; 12(1): 1–35. 3572272 10.1093/jmp/12.1.1

[42] Iloh G. U. P., Ofoedu J. N., Njoku P. U., Odu F. U., Ifedigbo C. V. and Iwuamanam K. D. Evaluation of patients' satisfaction with quality of care provided at the National Health Insurance Scheme clinic of a tertiary hospital in South-Eastern Nigeria. *Nigerian journal of clinical practice*, 2013; 15(4): 469–474. 10.4103/1119-3077.104529 23238200

[43] Eke C. B., Ibekwe R. C., Muoneke V. U., Chinawa J. M., Ibekwe M. U., Ukoha O. M. and Ibe B. C. End-users’ perception of quality of care of children attending children’s outpatients clinics of University of Nigeria Teaching Hospital Ituku-Ozalla Enugu. *BMC Research Notes*, 2014; 7(1): 800–805. 25399201 10.1186/1756-0500-7-800 PMC4247624

