# Supplementary material for: Overdependence on For-Profit Pharmacies: A Descriptive Survey of User Evaluation of Medicines Availability in Public Hospitals in Selected Nigerian States
Source: PLoS One. 2016 Nov 3;11(11):e0165707. doi: 10.1371/journal.pone.0165707 (PMC5094727; doi:10.1371/journal.pone.0165707)
Supplement: S1 File — (DOCX) [file pone.0165707.s001.docx]

sMEDICINES AVAILABILITY IN NIGERIA

(MAiN)

**Questionnaire**

**SECTION A: Socio-Demographic Data**

| S/N | QUESTION | RESPONSES | CODE |
| --- | --- | --- | --- |
| 1 | What is your Gender? | Male | 1 |
|  |  | Female | 2 |
| 2 | How old are you? | ................................... |  |
| 3 | What is your current marital status? | Single | 1 |
|  |  | Married | 2 |
|  |  | Divorced | 3 |
|  |  | Separated | 4 |
|  |  | Widowed | 5 |
|  |  | Others specify____________ | 58 |
| 4 | What is your educational level? | Primary | 1 |
|  |  | Secondary | 2 |
|  |  | NCE/OND | 3 |
|  |  | Degree/HND | 4 |
|  |  | Postgraduate | 5 |
|  |  | Others__________________ |  |
| 5 | What is your religion? | Christianity | 1 |
|  |  | Islam | 2 |
|  |  | Others __________________ | 58 |
| 6 | What is your present occupation? | __________________________________________________________________________ |  |
| 7 | What is the name of your place of work | ________________________ |  |
| 8 | Approximately, how much do you earn per month? | ______________________ |  |
| 9 | Are you presently using the National Health Insurance Scheme for your health care? | No | 0 |
|  |  | Yes | 1 |
| 10 | Is there any other health insurance scheme that you are covered in? | No | 0 |
|  |  | Yes | 1 |

**Section B: Quality of Care in Government Hospitals**

|  |  | RESPONSES | CODE |
| --- | --- | --- | --- |
| 11 | Which health care facilities are available within your locality where you live?  **(Multiple options allowed)** | None | 0 |
|  |  | Government hospital | 1 |
|  |  | Private hospital | 2 |
|  |  | Spiritual Healer | 2 |
|  |  | Folk Healer | 3 |
|  |  | Drug store (chemist) | 4 |
|  |  | Patent medicine vendor | 5 |
|  |  | Others specify.............. | 11 |
| 12 | When a member of your household falls sick, where do they usually go for treatment?  **(Multiple options allowed)** | Government hospital | 1 |
|  |  | Private hospital | 2 |
|  |  | Spiritual Healer | 2 |
|  |  | Folk Healer | 3 |
|  |  | Drug store (chemist) | 4 |
|  |  | Patent medicine vendor | 5 |
|  |  | None of the above | 6 |
|  |  | Others ________________ | 11 |
| 13 | **If none of the sources above**, what do you usually do? | ______________________________________________ |  |
| 14 | Why do you or your family member prefer to do this instead of going to the hospital? | _____________________________________________________________________ |  |
| 15 | Are medicines often available for treatment of the sick person in that hospital where you or your relatives go? | No | 0 |
|  |  | Yes | 1 |
| 16 | Concerning medicine availability, which do you prefer? | Government | 1 |
|  |  | Private | 2 |
|  |  | Chemist | 3 |
|  |  | Pharmacy | 4 |
|  |  | Any medicine store | 5 |
| 17 | What do you think of medicines in a government hospital? | Low standard | 1 |
|  |  | Average | 2 |
|  |  | High standard | 3 |
|  |  | Others _________________ | 11 |
| 18 | Are medicines usually available in the government hospitals that you have attended? | No, not at all | 1 |
|  |  | Yes, all the medicines I need | 2 |
|  |  | Yes, most of the medicines | 3 |
|  |  | Yes, some of the medicines | 4 |
| 19 | What will you say concerning availability of medicines in the government hospitals you have attended? |  |  |
| 20 | How would you rate the quality of medicines given in such a government hospital | Poor | 1 |
|  |  | Bad | 2 |
|  |  | Good | 3 |
|  |  | Very good | 4 |
| 21 | Do you usually find all you medicines in the hospital or you must buy some outside the hospital? | Always buy all in hospital | 1 |
|  |  | Some in the hospital | 2 |
|  |  | Buy all outside the hospital | 3 |
|  |  | Buy some outside the hospital | 4 |
| 22 | How do these medicines enhance the quality of care and health service delivery at government health facilities | _______________________________________________________________________ |  |

**Section C: Reasons for Using Private Pharmacies**

| 23 | For the illness that you are buying drugs, did you go to the hospital first before coming to the pharmacy? | No | | | 0 |
| --- | --- | --- | --- | --- | --- |
|  |  | Yes | | | 1 |
| 24 | If yes, which hospital did you go? |  | | |  |
| 25 | Is it a government or private hospital? | Government | | | 1 |
|  |  | Private | | | 2 |
| `26 | Are you buying these medicines for yourself or someone else? | For myself | | | 1 |
|  |  | For someone, specify __________ | | | 2 |
| 27 | Are you or the person you are buying drugs for on admission in the hospital? | No | | |  |
|  |  | Yes | | |  |
| 28 | Which drugs are you buying from here? | __________________________________________________________________________________________ | | |  |
| 29 | For which kind of health problem are you buying the medicines? | __________________________________________________________ | | |  |
| 30 | Are you here at the pharmacy with your hospital prescription or you are just buying medicine on your own? | Have hospital prescription | | | 1 |
|  |  | Buying on my own | | | 2 |
|  |  | Have doctor’s prescription but on ordinary paper | | | 3 |
| 31 | **If you have a hospital prescription,** why did you not get the medicines at the hospital? PLEASE DO NOT READ OPTIONS TO RESPONDENTS | | | |  |
|  | a) Medicines were not available there | | | No | Yes |
|  | b) There were too many people in line | | | No | Yes |
|  | c) The process of getting medicines in the hospital was too long | | | No | Yes |
|  | d) Did not have money to buy all the medicine but I can buy part here | | | No | Yes |
|  | e) In this pharmacy I can buy on credit | | | No | Yes |
|  | f) The owner of this pharmacy is a friend/relative | | | No | Yes |
|  | g) The medicines here are better than those in the hospital | | | No | Yes |
|  | h) I was referred here from the hospital | | | No | Yes |
|  | i) it is more costly to buy medicines in the hospital | | | No | Yes |
|  | j) It takes a longer time to get medicines at the hospital | | | No | Yes |
|  | k) Only some of my medicines were available in the hospital | | | No | Yes |
|  | l) Attitude of health care workers in the hospital | | | No | Yes |
|  | m) I prefer private pharmacy where all my medicines will be available | | | No | Yes |
|  | n) Others. ............................................. | | |  |  |
|  | o) Others................................................ | | |  |  |
|  | p) Others............................................. | | |  |  |
| 32 | **If you do not have a hospital prescription:**  a) Why did you not go to the hospital? | | ____________________________________ | |  |
|  | b) How did you know what is wrong with you? | | ____________________________________ | |  |
|  | c) How did you know which medicine to buy | | ____________________________________ | |  |
|  | d) How would you know how to take the medicines? | | ____________________________________ | |  |
| 33 | **If you had been to the hospital before coming here:**  a) How much money did you spend in the hospital? | | _________________ | |  |
|  | b) How much of what you spent was for medicine | | _________________ | |  |
|  | c) How much have you spent in this store to buy medicines? | | _________________ | |  |
| 34 | **If you did not first go to the hospital,** how much have you spent to buy your medicines here? | | _________________ | |  |

**Medicines Availability and Health Seeking Behaviour**

| 35 | If medicines are not available at the government hospital, will it affect your ability to seek health when the need arises? | No | 0 |
| --- | --- | --- | --- |
|  |  | Yes | 1 |
| 36 | **If yes,** please explain why this is so | ______________________________________ |  |
| 37 | **If No**, why does it not | ______________________________________ |  |
| 38 | Does the non-availability of medicines at the hospitals influence your choice of where to seek health care? | No  Yes | 0  1 |
| 39 | What other factors besides the non-availability of medicines affects your readiness to seek care in the hospital? | _________________________________________________________ |  |

**Section D: Practice of Self-Medication**

| 40 | Have you ever used medicines without prescription from a doctor or nurse? | No  Yes | 0  1 |
| --- | --- | --- | --- |
| 41 | **If Yes**, why? | ........................................................................................................................................................................................ |  |
| 42 | **If yes,** how often do you do this? | Whenever I am sick | 1 |
|  |  | When I have no money to go to hospital | 2 |
|  |  | When the illness is not a serious one | 3 |
|  |  | When I want to use an old prescription | 4 |
|  |  | Others ................................ |  |
| 43 | **If No**, why do you not use medicines without prescription? | ....................................................................................................................................................................................... |  |
| 44 | When you fall sick, do you usually take medicines by yourself or do you seek the opinion of a medical expert to prescribe medicines to you | Take medicine myself |  |
|  |  | Seek help from doctor |  |
|  |  | Do nothing |  |

**Section E: Preferred Sources of Medicines**

| 45 | Where do you buy your medicines you use when you fall ill? | …………………………………………………………………………………… |  |
| --- | --- | --- | --- |
| 46 | Why do you prefer to buy the medicines from the sources you mentioned? | ……………………………………………………………… |  |
| 47 | How long have you been buying medicines from this place? | ________________________________ |  |
| 48 | Why do you prefer to purchase the medicines you take from this source | ……………………………………………………………… |  |
| 49 | Do you have any relationship with the owner/attendant in the place you purchase the medicines? | No | 0 |
|  |  | Yes | 1 |
| 50 | If yes, of what benefit is this relationship to your ability to pay for medicines in this place? | _________________________________________________________ |  |
| 51 | Can you obtain medicines from this store on credit? | No | 0 |
|  |  | Yes | 1 |
| 52 | If yes, is that the reason why you prefer the pharmacy to buying medicines in the hospital? | No | 0 |
|  |  | Yes | 1 |
| 53 | Is this the only place in the community where you can purchase the medicines? | No | 0 |
|  |  | Yes | 1 |
| 54 | How good do you think the medicines sold in this place are? | Bad | 1 |
|  |  | Good | 2 |
|  |  | Very good | 3 |
